# Colistin, Meropenem–Vaborbactam, Imipenem–Relebactam, and Eravacycline Testing in Carbapenem-Resistant Gram-Negative Rods: A Comparative Evaluation of Broth Microdilution, Gradient Test, and VITEK 2

**DOI:** 10.3390/antibiotics13111062

**Published:** 2024-11-08

**Authors:** Patrick Forstner, Lisa Fuchs, Josefa Luxner, Andrea Grisold, Ivo Steinmetz, Karl Dichtl

**Affiliations:** Diagnostic and Research Institute of Hygiene, Microbiology and Environmental Medicine, Medical University of Graz, 8010 Graz, Austria; patrick.forstner@medunigraz.at (P.F.);

**Keywords:** VITEK 2 AST-XN24, gradient test, *Pseudomonas aeruginosa*, *Acinetobacter baumannii* complex, Enterobacterales, carbapenem-resistant organism, colistin, eravacycline, meropenem–vaborbactam, imipenem–relebactam

## Abstract

Objectives. This study aimed to evaluate and compare the performance of different assays for antimicrobial susceptibility testing (AST) and minimum inhibitory concentration (MIC) determination for reserve antibiotics in carbapenem-resistant Enterobacterales (CREs), *Pseudomonas aeruginosa* (CRPAs), and *Acinetobacter baumannii* (CRABs). Methods. An analysis was conducted on 100 consecutive isolates: 50 CREs, 35 CRPAs, and 15 CRABs. Sensititre broth microdilution was used as a reference standard to evaluate the performance of VITEK 2 card AST-XN24 (bioMérieux), the respective gradient tests (bioMérieux), and UMIC colistin broth microdilution test strips (Bruker Daltonics). Errors, essential agreement (EA), and categorical agreement of MICs for colistin (COL), meropenem–vaborbactam (MVB), imipenem–relebactam (IRL), and eravacycline (ERV) were assessed. Results. The agreement between both of the COL broth microdilution (BMD) methods was perfect (100/100). The gradient test and VITEK 2 analysis yielded comparable EA rates (92/100 and 72/79, respectively), with the latter not registering any very major errors (VMEs). The MVB gradient test achieved EA in 66 of 85 isolates and VITEK 2 in 70/85. For IRL, EA was reached in 69 and 64 of 85 cases by gradient test and VITEK 2 analysis, respectively. The ERV gradient test yielded false results in nearly all (12/15) CRABs but achieved EA in 46 of 50 CREs. The VITEK system recorded EA for ERV in 60 of 65 isolates. Conclusions. We observed substantial variability in the measured MICs between BMD and the alternative methods. In only a few constellations, VITEK 2 or gradient tests could substitute the reference method. BMD is the method of choice for COL analysis, with VITEK 2 representing an alternative method for CRPA testing. Alternative methods for MVB did not provide reliable results, except for Enterobacterales, when tested with the gradient test. However, resistant results need to be confirmed by BMD. Only BMD can be used for IRL MIC determination. VITEK 2 was mostly accurate in measuring ERV MICs, while the corresponding gradient test yielded reliable results exclusively in CREs. It is essential that laboratories are aware of which testing method provides reliable results for each combination of microorganisms and reserve antibiotics.

## 1. Introduction

In recent decades, bacterial antimicrobial resistance (AMR) has become one of the leading threats to public health. For 2019, it was estimated that 1.27 million global deaths were directly attributable to AMR, and a further 3.68 million were associated with AMR [1]. In 2050, deaths are expected to reach 1.91 and 8.22 million, respectively [2]. AMR will lead to global economic losses of up to USD 3.4 trillion annually by 2030, and up to USD 6.1 trillion annually by 2050, while also leading to a pronounced increase in extreme poverty as the economies of low-income countries are disproportionally affected [3]. In light of this, there is an urgent need for the widespread availability of newly developed, affordable antimicrobial substances, along with well-defined methods and guidelines for laboratories to advise on their use.

Carbapenems possess the broadest spectrum of antibacterial activity within the group of β-lactam antibiotics and are therefore often considered the last resort for standard therapy [4]. However, their widespread use has led to the emergence of carbapenemases, β-lactamases capable of hydrolyzing not only carbapenems but, in many cases, all β-lactam antibiotics [4,5]. At present, carbapenem-resistant organisms (CROs) take prominent places on the World Health Organization’s (WHO) Bacterial Priority Pathogens List [6]. Carbapenem-resistant *Pseudomonas aeruginosa* (CRPA) is a “high-priority pathogen”, while carbapenem-resistant Enterobacterales (CREs) and carbapenem-resistant *Acinetobacter baumannii* (CRABs) are classified in the highest category as “critical priority pathogens” [6].

Only few treatment options remain in CRO: the polymyxin derivative colistin (COL), developed in the 1950s, acts as a detergent, disrupting the outer membrane of Gram-negative bacteria. The novel combination drugs imipenem–relebactam (IRL) and meropenem–vaborbactam (MVB) add a novel β-lactamase inhibitor to the carbapenemase [7,8,9]. Eravacycline (ERV) is a novel tetracycline with activity against a wide range of bacteria, including CREs and CRABs [9,10,11,12,13].

Previous investigations on the in vitro testing for these reserve antibiotics relied on the gold standard of antimicrobial susceptibility testing, i.e., broth microdilution (BMD) [8,14,15], which is also the recommended method by EUCAST [16]. Although offering the best reproducibility, this method for antimicrobial susceptibility testing (AST) is rarely used in routine laboratories since it is labor-intensive and expensive. Instead, alternative methods for the determination of minimum inhibitory concentrations (MICs) are typically employed, particularly gradient tests and automated AST systems. These systems, however, only serve as a surrogate and cannot replace BMD; the VITEK 2 system, often misinterpreted as a BMD method, only measures few distinct MIC benchmarks and matches the resulting phenotypes with its phenotype database to give a guess of the expected MICs. Gradient tests, on the other hand, depend on the ability of the specific antibiotic substance to diffuse into the agar, and they are not available for all combinations of bacteria/antibiotics. Yet, when it comes to reserve antibiotics, the literature evaluating their performance and limitations is scarce. This is particularly true for reserve antibiotic testing in CROs, although patients suffering from CRO infections are arguably the only group that benefits from these test results.

For validation and evaluation, according to the international standards ISO 20776-1 [17] and ISO 20776-2 [18], it is necessary to compare other susceptibility testing methods to BMD. Hence, the objective of this in vitro study was to evaluate a commercially available automated AST platform and the respective commercially available gradient tests for the MIC determination of COL, ERV, IRL, and MVB, as well as UMIC test strips for COL against BMD as a reference standard using CRE, CRPA, and CRAB isolates.

## 2. Results

### 2.1. Colistin

**Figure 1:**
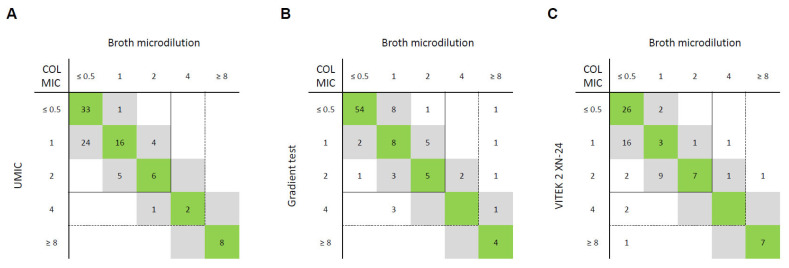
Correlation between colistin (COL) minimum inhibitory concentrations (MICs; in mg/L) of reference method (broth microdilution) and (**A**) UMIC broth microdilution, (**B**) gradient test, and (**C**) VITEK 2 card AST-XN24 for 100 Gram-negative bacterial isolates. MICs within essential agreement (within ±1 dilution of reference MIC) are highlighted in grey, and MICs identical to reference MICs are highlighted in green. EUCAST breakpoints are shown as lines (continuous lines for Enterobacterales and *Acinetobacter baumannii* complex: susceptible ≤ 2 mg/L, resistant > 2 mg/L; dotted lines for *Pseudomonas aeruginosa*: susceptible ≤ 4 mg/L, resistant > 4 mg/L).

COL resistance rates were 16% for CREs (8/50), 3% for CRPAs (1/35), and 0% (0/15) for CRABs in our consecutive cohort (Supplementary Table S1). EA and CA between the two BMD methods, i.e., UMIC and Sensititre Susceptibility Plate, were perfect (100%) (Figure 1).

In the CREs group, the gradient test yielded EA in 96% (48/50) and CA in 94% (47/50) of isolates. Three VMEs were recorded, one being *Serratia marcescens*, which made it easy to identify the error due to the intrinsic resistance of this species. Gradient test EA and CA for CRPA isolates were 83% (29/35) and 97% (34/35), respectively, while recording five mEs and one VME. In CRAB isolates, the gradient test achieved perfect (15/15) EA and CA.

Due to the technical limitations of the VITEK 2 system, no colistin MICs can be obtained for *Enterobacter* spp., which reduced the number of CRE isolates to 40. In these, the EA and CA rates were both 93% (37/40). One mE, one ME, and two VMEs were recorded. Notably, both VMEs occurred again in *S. marcescens*, which supports the easy identification of the error. EA and CA for CRPA isolates were 96% (23/24) and 100% (24/24), respectively, while only recording one mE. VITEK analysis achieved EA in 80% (12/15) and CA in 87% (13/15) of CRAB isolates. Here, one mE and two MEs were observed.

The results of bias calculation according to ISO 20776-2 [18] for all combinations of method and germ group are presented in Table 1.

### 2.2. Meropenem–Vaborbactam

MVB resistance rates were 56% in CREs (28/50) and 40% in CRPAs (14/35) (Supplementary Table S1).

**Figure 2:**
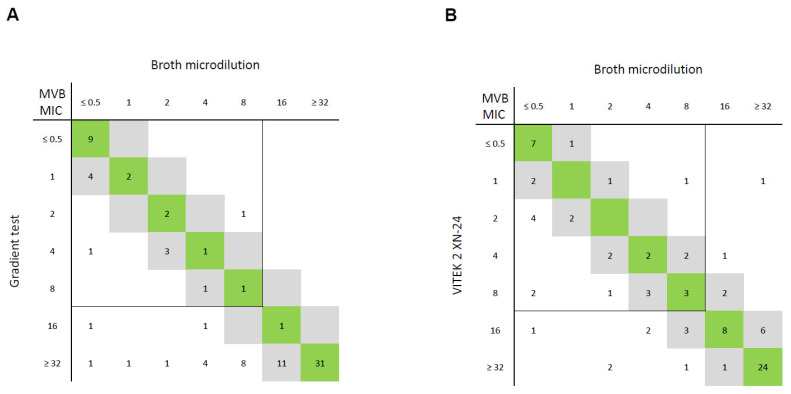
Correlation between meropenem–vaborbactam (MVB) minimum inhibitory concentrations (MICs; in mg/L) of reference method (broth microdilution) and (**A**) gradient test and (**B**) VITEK 2 card AST-XN24 for 85 Gram-negative bacterial isolates. MICs within essential agreement (within ±1 dilution of reference MICs) are highlighted in grey, and MICs identical to reference MICs are highlighted in green. EUCAST breakpoints for Enterobacterales and *Pseudomonas aeruginosa* are shown as lines (susceptible ≤ 8 mg/L, resistant > 8 mg/L).

The gradient test achieved EA in 86% (43/50) and CA in 90% (45/50) of CREs (Figure 2). Besides two mEs, 10% of CRE results (5/50) were MEs, i.e., reported as false-resistant. FOR CRPAs, 66% (23/35) EA and 66% CA were recorded. All deviations represented MEs (12/35).

VITEK 2 analysis of the CRE isolates demonstrated EA in 72% (36/50), recording seven mEs, five MEs, and two VMEs. Although CRPA testing reached 97% EA (34/35), the recorded number of MEs (4/35) and VMEs (2/35) did not differ significantly from those detected in CREs.

### 2.3. Imipenem–Relebactam

In total, 25 out of 50 CRE isolates (50%) and 14 out of 35 CRPA isolates (40%) displayed MICs below the EUCAST breakpoints for Enterobacterales and *P. aeruginosa* (Supplementary Table S1) [19].

**Figure 3:**
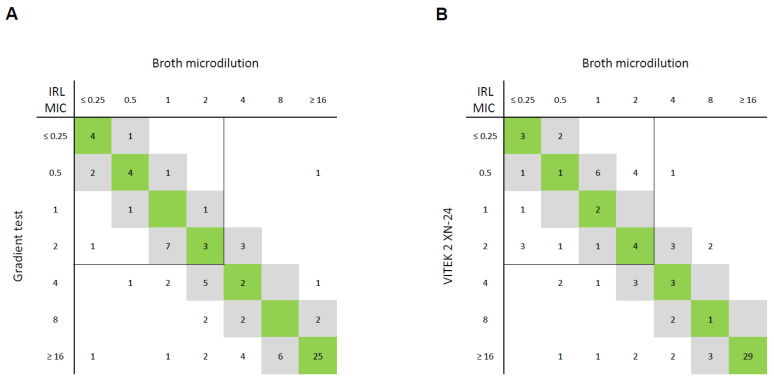
Correlation between imipenem–relebactam (IRL) minimum inhibitory concentrations (MICs; in mg/L) of reference method (broth microdilution) and (**A**) gradient test and (**B**) VITEK 2 card AST-XN24 for 85 Gram-negative bacterial isolates. MICs within essential agreement (within ±1 dilution of reference MICs) are highlighted in grey, and MICs identical to reference MICs are highlighted in green. EUCAST breakpoints for Enterobacterales and *Pseudomonas aeruginosa* are shown as lines (susceptible ≤ 2 mg/L, resistant > 2 mg/L).

The gradient test demonstrated EA in 78% (39/50) and CA in 81% (38 of 47 isolates, for which EUCAST breakpoints apply) of CREs (Figure 3). While only one mE occurred, ten MEs and three VMEs were recorded. For CRPAs, the EA and CA rates were 86% (30/35) and 74% (26/35), respectively, amounting to seven MEs and two VMEs.

VITEK 2 analysis yielded an EA rate of 72% (36/50) and a CA rate of 77% (36/47) for CREs, while recording four mEs and nine MEs. In the CRPA group, 80% (28/35) and 83% (29/35) of isolates reached EA and CA, respectively. A total of 4 and 6 out of 35 VITEK 2 results for CRPA isolates featured mEs and VMEs, respectively.

### 2.4. Eravacycline

In total, 38 out of 50 CRE isolates (76%, including all 5 *E. coli*) and all 15 CRABs (100%) displayed MICs below 1 mg/L, representing the EUCAST breakpoint for *E. coli* (Supplementary Table S1) [19].

**Figure 4:**
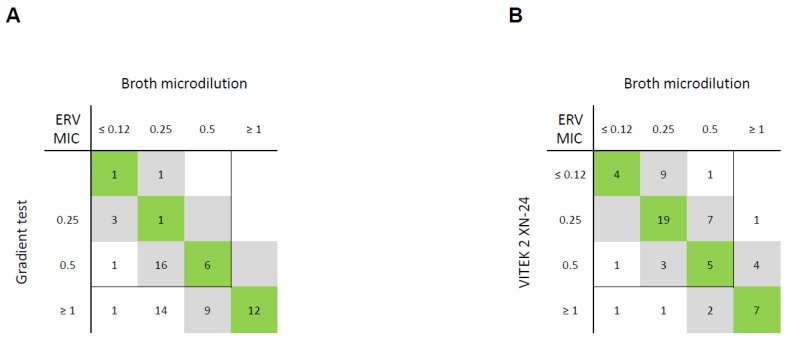
Correlation between eravacycline (ERV) minimum inhibitory concentrations (MICs; in mg/L) of reference method (broth microdilution) and (**A**) gradient test and (**B**) VITEK 2 card AST-XN24 for 65 Gram-negative bacterial isolates. MICs within essential agreement (within ±1 dilution of reference MICs) are highlighted in grey, and MICs identical to reference MICs are highlighted in green. EUCAST breakpoints for *E. coli* are shown as lines (susceptible ≤ 8 mg/L, resistant > 8 mg/L).

Using the ERV gradient test, 92% (46/50) of CRE isolates exhibited EA, whereas only 20% (3/15) of CRAB isolates achieved the same (Figure 4). The VITEK system, however, recorded EA in all 15 (100%) CRAB isolates while also reaching EA in 90% (45/50) of CRE isolates.

Due to this fact, that clinical breakpoints for ERV have only been established for *E. coli* so far; differentiation between mEs, MEs, and VMEs was only possible for a small subset of this study [19]. In *E. coli* isolates, the gradient test achieved EA in 100% (5/5) while still recording one ME. In contrast, analysis via VITEK 2 reached EA in 80% of cases, with the sole error being an mE.

## 3. Discussion

In this study, significant differences in the performance of two widely used commercially available AST methods in comparison to the reference standard of BMD were demonstrated. While some combinations of methods, antibiotic substances, and organisms demonstrated a high degree of correlation, others resulted in worryingly high error rates.

For COL, highly consequential VMEs were observed for VITEK 2 and gradient test analysis. However, in CREs and CRPAs, the VITEK 2 system was less error-prone than the gradient test, only leading to one ME and one VME, in contrast to four VMEs from the gradient test. It should be noted, however, that three of these five VMEs occurred in isolates belonging to species with primary resistance to COL, which are therefore unlikely to be falsely reported as susceptible. In contrast to the CREs and CRPAs, the gradient test delivered perfect results in our 15 highly COL-susceptible CRAB isolates. The two BMD methods, UMIC and Sensititre, demonstrated perfect concordance for all the included isolates.

While some studies reported VITEK 2 COL testing to be reliable [20,21], others were unable to reproduce these findings [22,23,24]. For example, contrary to our findings, Rout et al. found that the VITEK 2 system produced the most errors in *A. baumannii* (27.8% of VME and 7.9% of ME) [24]. EUCAST reported the frequent occurrence of VMEs in semi-automated colistin methods in 2016 [25] and published an extensive evaluation of COL gradient tests in 2017 [9], subsequently recommending the use of BMD for COL MIC determination [19]. The findings in this study support the conclusion that only BMD leads to reliable results.

MVB testing via VITEK 2 resulted in low EA (72%), with nine MEs and four VMEs, which limits its usability. While gradient tests yielded a comparable EA (78%), 17 MEs (17/85, 20%) occurred, since this method consistently delivered elevated MICs compared to the reference standard. CRPAs accounted for over two thirds (12/17) of these MEs. The VITEK 2 analysis resulted in nine MEs and four VMEs, which accounted for 15% of the tested isolates.

The only study evaluating MVB gradient tests came to the conclusion that the bioMérieux gradient test leads to an unacceptably high VME rate of 7% when using EUCAST breakpoints, while being accurate and reproducible when using CLSI/FDA breakpoints [26]. Contrarily, in this study, consistently elevated MICs occurred, and therefore MEs, but no VMEs, were found. The VITEK 2 performance of MVB in Enterobacterales and *P. aeruginosa* was reported to be good when considering EUCAST breakpoints, with 97% EA, 99% CA, 2% VMEs, and 1% MEs [7], which is also in stark contrast to this study’s findings.

In 18% of CREs and 26% of CRPAs, the IRL gradient test yielded false-resistant or false-susceptible results. The total error rate (mEs + MEs + VMEs) was even higher using VITEK 2 (32%) compared with the gradient test (25%). Interestingly, CREs were responsible for all eleven MEs (13%), while CRPAS accounted for all six VMEs (7%).

Previous research has found that the IRL gradient tests produce comparable results to BMD in Enterobacterales and *Pseudomonas aeruginosa* [27]. These findings were not reproduceable in this Austrian cohort; false-resistant or false-susceptible results were recorded in 24% (CREs) and 26% (CRPAs), respectively. Even though we are not aware of other studies investigating VITEK 2 performance for IRL testing, our results discourage the use of AST card XN-24 for this purpose.

In CREs, EA for ERV testing was ≥ 90% for both the gradient test and VITEK 2. In CRABs, 100% EA by VITEK 2 was contrasted by only 20% EA in the gradient test.

The only previously published evaluation of ERV gradient tests was conducted by the manufacturer itself and reported EA with BMD in 99% of Enterobacteriaceae, superseding our EA rate for Enterobacteriaceae, which was 91% (41/45) after the exclusion of other Enterobacterales [28]. To date, no research on the performance of ERV on the VITEK 2 system has been published.

For the verification of new methods, the Clinical and Laboratory Standards Institute (CLSI) recommends the following criteria: EA and CA rates > 90%, an ME rate < 3%, and a VME rate < 1% [29]. In our study, these criteria were only met for COL: UMIC for all organism groups, VITEK 2 XN-24 for CREs and CRPAs, and the gradient test for CRABs.

This study is not without limitations. From a methodological perspective, it is unfavorable that not all tests were conducted on the same day. The selection of isolates might under-represent relevant species and resistance mechanisms. However, the inclusion of consecutive isolates recovered in a diagnostic laboratory depicts the epidemiology our routine laboratory is facing. We think that this also applies to the decision to exclude carbapenem-susceptible isolates, which usually do not require testing for reserve antibiotics.

To our knowledge, this is the most comprehensive study to date evaluating the performance of different methods for MIC determination of multiple reserve antibiotics in highly resistant Gram-negative isolates. With the wide range of different CROs used in this study, it was possible to investigate the performance of the different commercially available AST methods. The results can help laboratories to select a test system that is accurate and meets their needs. In contrast to previous research, no error adjustment protocols were used to reduce ME and VME rates by removing the ME/VME categorization from the isolates, of which the measured MICs deviated by only one titre dilution (still in EA) [7,28]. This might explain some of the differing results and conclusions. However, most of the detected differences can presumably be attributed to our isolate collection, which is characterized by carbapenem resistance; multiresistant isolates increase the difficulty of AST, and the testing of carbapenem/β-lactamase inhibitor combinations at a fixed inhibitor concentration seems to be particularly error-prone. Deviations of adjusted results from original results are summarized in Supplementary Table S2.

The results of the COL testing suggest that—with some limitations—VITEK 2 card AST-XN24 can be used, though BMD is clearly the method of choice. While this study was still being conducted, bioMérieux withdrew the test from the market. According to the obtained data, the MVB gradient test should only be used on Enterobacterales, and all resistant results should be confirmed by BMD. This study discourages using the VITEK 2 system for MVB MIC determination. Only BMD should be used for IRL MIC determination, as both the gradient test and VITEK 2 were highly inaccurate. For ERV, the gradient test delivered reliable results for CREs but not for CRABs, while VITEK 2 analysis proved to be mostly accurate for both groups.

More research needs to be performed on larger cohorts of non-wildtype isolates, especially focusing on single combinations of bacterial species and antibiotic agents.

## 4. Methods

A total of 100 pre-characterized, consecutive, and non-duplicate isolates of carbapenem-resistant Gram-negative rods detected in our routine bacteriologic laboratory between 2018 and 2024 were included. The collection comprised 50 isolates of Enterobacterales, 35 isolates of *P. aeruginosa,* and 15 isolates of the *A. baumannii* complex. An overview of all isolates is provided in Table 2. A detailed characterization of each isolate can be found in Supplementary Table S3.

All isolates were stored at −80 °C. Before initiating the study, the isolates were unthawed, manually inoculated onto Columbia blood agar (ref. 254071; Becton Dickinson, Franklin Lake, NJ, USA), and incubated overnight at 35 ± 1 °C. Pure isolates were passaged once in order to ensure that only fresh colonies were used for further analysis.

Antimicrobial susceptibility testing was performed using VITEK 2 card AST-XN24 (ref. 424351; bioMérieux, Marcy-l’Étoile, France) on a VITEK 2 XL device (bioMérieux), UMIC Colistin BMD (ref. UM-COL-040, Bruker Daltonics, Bremen, Germany), Sensititre Susceptibility Plates (ref. YEUMDRXXF; TREK Diagnostic Systems, East Grinstead, UK), and gradient tests manufactured by bioMérieux for colistin (ref. 537300), meropenem-vaborbactam (ref. 421563), imipenem-relebactam (ref. 420925), and eravacycline (ref. 421552). For all tests, inoculation and incubation were conducted according to the manufacturers’s protocols. For UMIC COL BMD and Sensititre BMD, the recommended Mueller Hinton broth was used (UMIC: ref. UM-MH-020, Bruker Daltonics; Sensititre: ref. YT3462, TREK Diagnostic Systems). The gradient tests were each placed on a separate Mueller Hinton agar (ref. 254081; Becton Dickinson). All media for BMD (including UMIC) and gradient testing were incubated in ambient atmosphere at 35 ± 1 °C for 18 ± 2 h.

MIC testing was performed with regards to the intrinsic resistance of the different organisms: Enterobacterales were tested for all substances, *A. baumannii* complex was tested for COL and ERV, while *P. aeruginosa* was tested for COL, IRL, and MVB. IRL and MVB were not tested in CRABs because the addition of relebactam and vaborbactam to carbapenem antibiotics does not improve the in vitro activity against carbapenem-non-susceptible isolates of *A. baumannii* [30,31,32]. ERV testing was waived for *P. aeruginosa* since previous studies found elevated MIC_50_ and MIC_90_ values, which are interpreted as intrinsic resistance [33,34,35]. The MIC ranges covered by the different methods are depicted in Supplementary Table S4. All BMD and gradient tests were read by two experienced microbiologists. In cases of disagreement, a third specialist was consulted for a decisive result.

Categorization of isolates as susceptible or resistant was inferred from the European Committee on Antimicrobial Susceptibility Testing (EUCAST) Breakpoint Tables for the interpretation of MICs and zone diameters, version 14.0 [19]. Essential agreement (EA = MICs within the range of ±1 dilution), categorical agreement (CA = correct interpretation as susceptible or resistant according to reference method), minor errors (mE = no EA, but CA), major errors (ME = false resistance), very major errors (VME = false susceptibility), and bias (percentage of MICs higher than the reference MIC subtracted by the percentage of MICs lower than the reference MIC) were calculated according to ISO standard 20776-2:2021 [18] and EUCAST Breakpoint Tables version 14.0 [19].

## Figures and Tables

**Table 1:** Overview of the calculated bias per method and AST method.

		Enterobacterales	*Pseudomonas aeruginosa*	*Acinetobacter baumannii* Complex
Colistin	UMIC	+14%	+9%	+100%
	Gradient test	−8%	−20%	0%
	VITEK 2	+3%	+38%	+93%
Meropenem- vaborbactam	Gradient test	+32%	+57%	-
	VITEK 2	+18%	+6%	-
Imipenem- relebactam	Gradient test	+30%	+34%	-
	VITEK 2	+38%	+37%	-
Eravacycline	Gradient test	+58%	-	+93%
	VITEK 2	−14%	-	−47%

**Table 2:** Overview of the included isolates.

		Number of Detected Carbapenemases				
Organism	Number of Isolates	One	Two	Three	None	Carbapenemase Rate (%)
Enterobacterales	50	33	7	-	10	80
*Klebsiella pneumoniae*	20	14	6	-	-	100
*Enterobacter cloacae* complex	10	3	1	-	6	40
*Escherichia coli*	5	5	-	-	-	100
*Klebsiella aerogenes*	5	1	-	-	4	20
*Klebsiella oxytoca*	2	2	-	-	-	100
*Proteus mirabilis*	2	2	-	-	-	100
*Serratia marcescens*	2	2	-	-	-	100
*Citrobacter freundii*	2	2	-	-	-	100
*Klebsiella ornithinolytica*	1	1	-	-	-	100
*Providencia stuartii*	1	1	-	-	-	100
*Pseudomonas aeruginosa*	35	17	-	-	18	49
*Acinetobacter baumannii* complex	15	9	4	1	1	93

## Data Availability

All data are available from the corresponding author on reasonable request.

## Supplementary Materials

The following supporting information can be downloaded at: https://www.mdpi.com/article/10.3390/antibiotics13111062/s1, Table S1. Number and percentage of resistant results for each antibiotic substance and method for all groups of isolates; Table S2. Differences in rates of major and very major errors before and after error adjustment [7,19]; Table S3. Detailed characterization of all isolates (species, carbapenemase type, and method of carbapenemase detection). The NG-Test CARBA5 (NG Biotech, Guipry, France) was used for immunologic testing. For molecular testing, either the Genotyping CarbaResist microarray (Inter Array by fzmb, Langensalza, Germany) was performed in our laboratory, or molecular testing was conducted by the Austrian National Reference Center for Antimicrobial Resistance and Nosocomial Infections; Table S4. Summary of minimum inhibitory concentration (MIC) measurement ranges for the employed assays.
