# Supplementary material for: Colistin, Meropenem–Vaborbactam, Imipenem–Relebactam, and Eravacycline Testing in Carbapenem-Resistant Gram-Negative Rods: A Comparative Evaluation of Broth Microdilution, Gradient Test, and VITEK 2
Source: Antibiotics (Basel). 2024 Nov 8;13(11):1062. doi: 10.3390/antibiotics13111062 (PMC11591322; doi:10.3390/antibiotics13111062)
Supplement: Supplementary file 1 [file antibiotics-13-01062-s001.zip › Supplementary Table S1_241014_PF.pdf]

Substance	Test method	Enterobacterales Resistance rate		*Pseudomonas aeruginosa* Resistance rate		*Acinetobacter baumanii* complex Resistance rate	
**Colistin**	**Broth microdilution**	**8 /50**	**16 %**	**1 / 35**	**3 %**	**0 / 15**	**0 %**
	UMIC	8 / 50	16 %	1 / 35	3 %	0 / 15	0 %
	VITEK 2	7 / 40	18 %	1 / 24	4 %	2 / 15	13 %
	Gradient test	5 / 50	10 %	0 / 35	0%	0 / 15	0 %
**Meropenem - vaborbactam**	**Broth microdilution**	**22 / 50**	**44 %**	**21 / 35**	**60 %**		
	VITEK 2	25/ 50	50 %	23 / 35	66 %		
	Gradient test	27 / 50	54 %	33 / 35	94 %		
**Imipenem - relebactam**	**Broth microdilution**	**24 / 47**	**51 %**	**21 / 35**	**60 %**		
	VITEK 2	29 / 47	62 %	15 / 35	43 %		
	Gradient test	33 / 47	70 %	26 / 35	74 %		
**Eravacycline**	**Broth microdilution**	**0 / 5**	**0 %**				
	VITEK 2	0 / 5	0 %				
	Gradient test	1 / 5	20 %				
