# Supplementary material for: Colistin, Meropenem–Vaborbactam, Imipenem–Relebactam, and Eravacycline Testing in Carbapenem-Resistant Gram-Negative Rods: A Comparative Evaluation of Broth Microdilution, Gradient Test, and VITEK 2
Source: Antibiotics (Basel). 2024 Nov 8;13(11):1062. doi: 10.3390/antibiotics13111062 (PMC11591322; doi:10.3390/antibiotics13111062)
Supplement: Supplementary file 1 [file antibiotics-13-01062-s001.zip › Supplementary Table S2_241014_PF.pdf]

Substance	Test method	Enterobacterales				*Pseudomonas aeruginosa*			
		Major errors		Very major errors		Major errors		Very major errors	
		before adjustment	after adjustment	before adjustment	after adjustment	before adjustment	after adjustment	before adjustment	after adjustment
**Meropenem - vaborbactam**	VITEK 2	-	-	-	-	4 / 35	1 / 35	2 / 35	0 / 35
	Gradient test	-	-	-	-	-	-	-	-
**Imipenem - relebactam**	VITEK 2	9 / 47	6 /47	-	-	-	-	5 / 35	3 / 35
	Gradient test	10 / 47	8 /47	2 / 47	1 / 47	7 / 35	4 / 35	2 / 35	0 / 35
**Eravacycline**	VITEK 2	-	-	-	-	-	-	-	-
	Gradient test	1 / 5	0 / 5	-	-	-	-	-	-
