# Supplementary material for: Colistin, Meropenem–Vaborbactam, Imipenem–Relebactam, and Eravacycline Testing in Carbapenem-Resistant Gram-Negative Rods: A Comparative Evaluation of Broth Microdilution, Gradient Test, and VITEK 2
Source: Antibiotics (Basel). 2024 Nov 8;13(11):1062. doi: 10.3390/antibiotics13111062 (PMC11591322; doi:10.3390/antibiotics13111062)
Supplement: Supplementary file 1 [file antibiotics-13-01062-s001.zip › Supplementary Table S3_240806_PF.pdf]

Isolate	Species	Carbapenemase(s)	Carbapenemase identification method
1	*Acinetobacter baumanii* complex	NDM-like + Oxa-40-like	Molecular Assay
2	*Acinetobacter baumanii* complex	Oxa-23-like	Molecular Assay
3	*Acinetobacter baumanii* complex	NDM-like + Oxa-23-like + Oxa-40-like	Molecular Assay
4	*Acinetobacter baumanii* complex	NDM-like + Oxa-40-like	Molecular Assay
5	*Acinetobacter baumanii* complex	Oxa-40-like	Molecular Assay
6	*Acinetobacter baumanii* complex	not detected	Molecular Assay
7	*Acinetobacter baumanii* complex	NDM-like + Oxa-23-like	Molecular Assay
8	*Acinetobacter baumanii* complex	Oxa-23-like	Molecular Assay
9	*Acinetobacter baumanii* complex	Oxa-23-like	Molecular Assay
10	*Acinetobacter baumanii* complex	Oxa-23-like	Molecular Assay
11	*Acinetobacter baumanii* complex	Oxa-23-like	Molecular Assay
12	*Acinetobacter baumanii* complex	Oxa-24-like	Molecular Assay
13	*Acinetobacter baumanii* complex	Oxa-23-like	Molecular Assay
14	*Acinetobacter baumanii* complex	Oxa-24-like	Molecular Assay
15	*Acinetobacter baumanii* complex	NDM-like + Oxa-40-like	Molecular Assay
16	*Pseudomonas aeruginosa*	IMP-like	Immunological Assay
17	*Pseudomonas aeruginosa*	VIM-like	Immunological Assay
18	*Pseudomonas aeruginosa*	not detected	Molecular Assay
19	*Pseudomonas aeruginosa*	not detected	Molecular Assay
20	*Pseudomonas aeruginosa*	VIM-like	Immunological Assay
21	*Pseudomonas aeruginosa*	not detected	Molecular Assay
22	*Pseudomonas aeruginosa*	not detected	Molecular Assay
23	*Pseudomonas aeruginosa*	not detected	Molecular Assay
24	*Pseudomonas aeruginosa*	not detected	Molecular Assay
25	*Pseudomonas aeruginosa*	not detected	Molecular Assay
26	*Pseudomonas aeruginosa*	IMP-like	Immunological Assay
27	*Pseudomonas aeruginosa*	not detected	Molecular Assay
28	*Pseudomonas aeruginosa*	VIM-like	Immunological Assay
29	*Pseudomonas aeruginosa*	VIM-like	Molecular Assay
30	*Pseudomonas aeruginosa*	not detected	Molecular Assay
31	*Pseudomonas aeruginosa*	VIM-like	Immunological Assay
32	*Pseudomonas aeruginosa*	not detected	Molecular Assay

33	*Pseudomonas aeruginosa*	IMP-like	Molecular Assay
34	*Pseudomonas aeruginosa*	VIM-like	Immunological Assay
35	*Pseudomonas aeruginosa*	not detected	Molecular Assay
36	*Pseudomonas aeruginosa*	not detected	Molecular Assay
37	*Pseudomonas aeruginosa*	IMP-like	Immunological Assay
38	*Pseudomonas aeruginosa*	not detected	Molecular Assay
39	*Pseudomonas aeruginosa*	VIM-like	Immunological Assay
40	*Pseudomonas aeruginosa*	VIM-like	Immunological Assay
41	*Pseudomonas aeruginosa*	VIM-like	Immunological Assay
42	*Pseudomonas aeruginosa*	not detected	Molecular Assay
43	*Pseudomonas aeruginosa*	not detected	Molecular Assay
44	*Pseudomonas aeruginosa*	not detected	Molecular Assay
45	*Pseudomonas aeruginosa*	VIM-like	Immunological Assay
46	*Pseudomonas aeruginosa*	VIM-like	Immunological Assay
47	*Pseudomonas aeruginosa*	IMP-like	Immunological Assay
48	*Pseudomonas aeruginosa*	VIM-like	Immunological Assay
49	*Pseudomonas aeruginosa*	not detected	Molecular Assay
50	*Pseudomonas aeruginosa*	not detected	Molecular Assay
51	*Klebsiella aerogenes*	Oxa-48 like	Molecular Assay
52	*Klebsiella pneumoniae*	Oxa-48-like	Molecular Assay
53	*Klebsiella pneumoniae*	NDM-like	Immunological Assay
54	*Klebsiella pneumoniae*	VIM-like + OXA-48-like	Immunological Assay
55	*Klebsiella pneumoniae*	NDM-like + Oxa-48-like	Molecular Assay
56	*Klebsiella aerogenes*	not detected	Molecular Assay
57	*Klebsiella pneumoniae*	NDM-like + Oxa-48-like	Molecular Assay
58	*Providencia stuartii*	NDM-like	Molecular Assay
59	*Enterobacter cloacae* complex	VIM-like	Molecular Assay
60	*Enterobacter cloacae* complex	not detected	Molecular Assay
61	*Proteus mirabilis*	VIM-like	Molecular Assay
62	*Enterobacter cloacae* complex	not detected	Molecular Assay
63	*Proteus mirabilis*	Oxa-48-like	Molecular Assay
64	*Klebsiella pneumoniae*	KPC-like	Molecular Assay
65	*Klebsiella oxytoca*	Oxa-48-like	Molecular Assay

66	*Klebsiella pneumoniae*	VIM-like	Molecular Assay
67	*Escherichia coli*	OXA-48-like	Molecular Assay
68	*Klebsiella pneumoniae*	KPC-like	Immunological Assay
69	*Klebsiella ornithinolytica*	Oxa-48-like	Molecular Assay
70	*Serratia marcescens*	VIM-like	Molecular Assay
71	*Klebsiella oxytoca*	VIM-like	Molecular Assay
72	*Serratia marcescens*	VIM-like	Molecular Assay
73	*Escherichia coli*	Oxa-48-like	Molecular Assay
74	*Klebsiella pneumoniae*	NDM-like	Immunological Assay
75	*Enterobacter cloacae* complex	VIM-like	Molecular Assay
76	*Klebsiella aerogenes*	not detected	Molecular Assay
77	*Escherichia coli*	OXA-48-like	Molecular Assay
78	*Enterobacter cloacae* complex	VIM-like	Molecular Assay
79	*Klebsiella pneumoniae*	NDM-like	Molecular Assay
80	*Citrobacter freundii*	Oxa-48	Molecular Assay
81	*Klebsiella pneumoniae*	NDM-like + Oxa-48-like	Immunological Assay
82	*Klebsiella pneumoniae*	NDM-like	Molecular Assay
83	*Enterobacter cloacae* complex	not detected	Molecular Assay
84	*Klebsiella pneumoniae*	NDM-like + Oxa-48-like	Molecular Assay
85	*Enterobacter cloacae* complex	not detected	Molecular Assay
86	*Enterobacter cloacae* complex	NDM-like + Oxa-48-like	Immunological Assay
87	*Escherichia coli*	KPC-like	Immunological Assay
88	*Klebsiella aerogenes*	not detected	Molecular Assay
89	*Enterobacter cloacae* complex	not detected	Molecular Assay
90	*Klebsiella pneumoniae*	NDM-like + Oxa-48-like	Molecular Assay
91	*Klebsiella pneumoniae*	Oxa-48-like	Immunological Assay
92	*Klebsiella pneumoniae*	OXA-48-like	Molecular Assay
93	*Klebsiella aerogenes*	not detected	Molecular Assay
94	*Klebsiella pneumoniae*	NDM-like	Molecular Assay
95	*Klebsiella pneumoniae*	VIM-like	Molecular Assay
96	*Citrobacter freundii*	VIM-like	Molecular Assay
97	*Enterobacter cloacae* complex	not detected	Molecular Assay
98	*Klebsiella pneumoniae*	Oxa-48-like	Molecular Assay

99	*Escherichia coli*	Oxa-48-like	Molecular Assay
100	*Klebsiella pneumoniae*	Oxa-48-like	Molecular Assay
