# Supplementary material for: Colistin, Meropenem–Vaborbactam, Imipenem–Relebactam, and Eravacycline Testing in Carbapenem-Resistant Gram-Negative Rods: A Comparative Evaluation of Broth Microdilution, Gradient Test, and VITEK 2
Source: Antibiotics (Basel). 2024 Nov 8;13(11):1062. doi: 10.3390/antibiotics13111062 (PMC11591322; doi:10.3390/antibiotics13111062)
Supplement: Supplementary file 1 [file antibiotics-13-01062-s001.zip › Supplementary Table S4_241014_PF.pdf]

| Test method | MIC range (mg/l) | |
| --- | --- | --- |
| | minimum | maximum |
| **Colistin** | | |
| Sensititre | 0.5 | 16 |
| UMIC | 0.06 | 64 |
| VITEK 2 | 0.125 | 8 |
| Gradient test | 0.016 | 256 |
| **Meropenem - vaborbactam** | | |
| Sensititre | 0.06 | 16 / 8 |
| VITEK 2 | 0.5 | 32 / 8 |
| Gradient test | 0.004 | 64 / 8 |
| **Imipenem - relebactam** | | |
| Sensititre | 0.06 | 8 / 4 |
| VITEK 2 | 0.25 | 16 / 4 |
| Gradient test | 0.002 | 32 / 4 |
| **Eravacycline** | | |
| Sensititre | 0.008 | 0.5 |
| VITEK 2 | 0.25 | 4 |
| Gradient test | 0.002 | 32 |
